# Long-standing pubic-related groin pain in professional academy soccer players: a prospective cohort study on possible risk factors, rehabilitation and return to play

**DOI:** 10.1186/s12891-021-04837-x

**Published:** 2021-11-17

**Authors:** Helge Eberbach, David Fürst-Meroth, Ferdinand Kloos, Magnus Leible, Valentin Bohsung, Lisa Bode, Markus Wenning, Schmal Hagen, Gerrit Bode

**Affiliations:** 1 Department of Orthopedic and Trauma Surgery, Medical Center - University of Freiburg, Faculty of Medicine, University of Freiburg, Hugstetter Str. 55, 79106 Freiburg, Germany; 2 Freiburg Youth Academy, Sports-Club Freiburg e.V., Schwarzwaldstr. 193, 79117 Freiburg, Germany; 3 Department of Orthopedic Surgery, University Hospital Odense, Sdr. Boulevard 29, 5000 Odense C, Denmark; 4 Sporthopaedicum Straubing, Bahnhofplatz 27, 94315 Straubing, Germany

**Keywords:** Pubic-related groin-pain, Risk factors, Rehabilitation, RTP, RTS

## Abstract

**Background:**

Despite being a common overuse entity in youth soccer, scientific data on risk factors, rehabilitation and return to play for long-standing pubic-related groin pain is still rare. The current prospective cohort study aims to evaluate potential risk-factors, propose a criteria-based conservative rehabilitation protocol and assess return-to-play outcomes among professional youth soccer players suffering from long-standing pubic-related groin pain.

**Methods:**

Male soccer players with long-standing (> 6 weeks) pubic-related groin pain from a professional soccer club’s youth academy were analyzed for possible risk factors such as age, team (U12 - U23), younger/older age group within the team, position and preinjury Functional movement score. All injured players received a conservative, standardized, supervised, criteria-based, 6-level rehabilitation program. Outcome measures included time to return to play, recurrent groin pain in the follow-up period and clinical results at final follow-up two years after their return to play.

**Results:**

A total of 14 out of 189 players developed long-standing pubic-related groin pain in the 2017/2018 season (incidence 7.4%). The average age of the players at the time of the injury was 16.1 ± 1.9 years. Risk factor analysis revealed a significant influence of the age group within the team (*p* = .007). Only players in the younger age group were affected by long-standing pubic-related groin pain, mainly in the first part of the season. Injured players successfully returned to play after an average period of 135.3 ± 83.9 days. Only one player experienced a recurrence of nonspecific symptoms (7.1%) within the follow-up period. The outcome at the 24-month follow-up was excellent for all 14 players.

**Conclusions:**

Long-standing pubic-related groin pain is an overuse entity with a markedly high prevalence in youth soccer players, resulting in a relevant loss of time in training and match play. In particular, the youngest players in each team are at an elevated risk. Applying a criteria-based rehabilitation protocol resulted in an excellent return-to-play rate, with a very low probability of recurrence.

**Trial registration:**

The trial was retrospectively registered under DRKS00016510 in the German Clinical Trials Register on 19.04.2021.

**Supplementary Information:**

The online version contains supplementary material available at 10.1186/s12891-021-04837-x.

## Background

Groin injuries are among the most common injuries in professional youth soccer and can cause the loss off considerable time from training and match play [1–3]. Recent epidemiological data suggests that approximately 10% of all youth soccer players sustain a groin injury each season [4, 5]. Groin pain can be caused by a wide variety of different pathological conditions, resulting in overlapping clinical symptoms [6–8].

Therefore, the Doha agreement about a broadly defined and clinically-based classification system of groin pain has been published in the last decade [9]. Long-standing pubic-related groin pain is an entity characterized with typical clinical history, physical findings and imaging morphology [10–12]. It is a noninfectious, inflammatory, chronic injury involving the symphysis, pubic rami and the surrounding soft tissue resulting from chronic overuse [10, 13, 14]. The clinical appearance is characterized by typically pubic-related pain during activity and local tenderness over the symphysis and pubic bone [11, 13]. MRI may show juxta-articular bone marrow edema in the adjacent pubis and possibly an irregular symphysis gap; accompanying edema in the surrounding muscles can also be observed [12, 15]. In CT-scans open pubic apophyses with stress-related physeal changes (widening, asymmetry and small rounded cyst-like expansions) can also be observed [16]. Commonly, it affects high-level athletes, in particular those who participate in sports that involve kicking, turning, twisting, cutting, pivoting, sprinting, rapidly accelerating and decelerating [8].

Anatomically important muscle groups, such as the adductors (especially the M. adductor longus) and the rectus abdominis muscle, which are firmly connected to the pubic bone and stabilize the anterior pelvis, are in close positional relationship in the pubic region [17, 18]. The intense opposite strain on the muscles, particularly in soccer, leads to repetitive microtrauma in the area of the tendinoperiosteal junction on the pubic bone [10]. Histological examinations biopsies in patients with long-standing pubic-related groin pain showed osteoblasts, new bone growth and neovascularization in the affected bone. The morphological correlate of the edema detected on MRI is most likely microstructural trabecular fractures, which occur due to antagonistic forces acting on the os pubis [19, 20].

In particular, young soccer players at the time of their largest increase in body height have an increased risk for overuse injuries around the groin [2, 5]. Decreases in flexibility and bone density during the growth spurt may result in greater vulnerability of the skeletal system [21, 22]. This vulnerability combined with the increase in training and game load can lead to long-standing pubic-related groin pain, which then can lead to the relevant loss of practice and play time for the injured player [10, 23, 24].

To date, reliable scientific data on risk factors for, rehabilitation of and return to play after long-standing pubic-related groin pain in professional youth soccer players is missing. Thus, diagnosis, treatment and return to play are challenging for the treating physiotherapists, athletic trainers and team physicians. The aim of this study was to evaluate the effect of age, player position and Functional movement score (FMS) for long-standing pubic-related groin pain in players in a professional youth soccer academy, implement a standardized nonsurgical therapeutic regimen and prospectively evaluate the outcome.

## Methods

### Patients

All elite male soccer players playing for a German first division soccer club youth academy (11 to 22 years) in the 2017/2018 season were included in this prospective study. Players suffering from long-standing pubic-related groin pain (Study Group) were compared to non-affected players (Control Group).

The inclusion criteria for the Study Group were as follows: anamnesis of exacerbating atraumatic groin pain during training or everyday life loads, positive clinical pubic-related groin pain signs and at least parasymphyseal pubic bone marrow edema on MRI.

Exclusion criteria were age > 22 years and other interfering pathologies resulting in groin pain, especially clinical findings indicating sportsman hernia; inguinal or femoral hernia; evidence of prostatitis or chronic urinary tract disease; pain of the vertebrae from the tenth thoracic segment to the fifth lumbar segment, including the facet joints and the sacroiliac joint; presence of malignant disease; clinical findings showing nerve entrapment of the ilioinguinal, genitofemoral, iliohypogastrical or lateral femoral cutaneous nerves; clinical or radiographic evidence of hip joint diseases; bursitis of the hip or groin region. The Control Group consisted of all players without specific long-standing pubic-related groin pain symptoms during the season in question. Ethical approval was obtained for this study, and written informed consent was acquired from all athletes before inclusion. For adolescents < 18 years, informed consent was acquired from their parents.

### Diagnostic criteria

At presentation, the diagnostic process started with recording the medical history and performing a clinical evaluation. Youth players suffering from long-standing pubic-related groin pain typically present with nonacute, load-dependent pain over the os pubis or symphyseal gap. Pain can be unilateral or bilateral, and it is typically exacerbated by running, kicking, hip adduction or flexion, and eccentric loads on the *M. rectus* abdominis and/or the adductors [11, 25].

Clinically, pressure pain over the os pubis, either as a result of deep palpation or even pain at rest, is the primary clinical sign [26, 27]. Due to the effect on the proximal origin of the adductor muscles, the adductor squeeze test at 0-, 45- and 90-degree hip flexion is frequently also positive [28]. Moreover, the patients were clinically tested for femoroacetabular impingement with the FABER and FADIR tests, and ultrasound was used in all patients to assist the physical examination and exclude a sportsman or inguinal hernia.

All athletes were additionally imaged using a 1.5 Tesla MRI (Magnetom Avanto; Siemens). MRI was performed using routine coronal, axial and sagittal T1-weighed and T2-weighted sequences as well as coronal and axial STIR (short tau inversion recovery) sequences to detect bone marrow edema [12]. Edema was graded as 1 (edema in the pubic bone unilateral or bilateral), 2 (edema in the pubic bone unilateral or bilateral, with participation of the periosseous tissue and/or muscles) and 3 (edema in the pubic bone, fluid in the symphysis gap and/or the periosseous muscles) according to the Krueger classification system [29]. All MRI scans were reviewed independently by two experienced orthopedic doctors (LB and FK), who were blinded to all clinical information of the study participants. In case of a different assessment, a collaborative consensus was reached.

In summary, the minimum criteria for inclusion in the study group were pubic-related groin pain > 6 weeks with exacerbation under load, pressure pain over the os pubis, bone marrow edema on MRI and the absence of other symptomatic pathologies of the groin.

### Analysis of risk factors

To determine the risk factors, the age, team (U-12 to U-23), age group within the particular team, position (goalkeeper/defender/midfielder/striker) and existing curricular preseason FMS score were recorded and compared to those of the players without diagnoses of long-standing pubic-related groin pain.

The Functional Movement Screen as developed by Gray Cook included “deep squat,” “hurdle step,” “in-line lunge,” “shoulder mobility test,” “active straight leg raise,” “trunk stability push-up,” and a “rotary stability test” [30, 31]. All items were performed 3 times, and the best trial was scored. A 4-point ranking system was used to evaluate the movement quality. The total FMS score was the summation of all 7 scores, resulting in a maximum of 21 points [32]. Two experienced examiners, the physiotherapist VB and the youth athletics coach ML, recorded the FMS score during the standardized preseason screening.

### Rehabilitation program

**Fig. 1:**
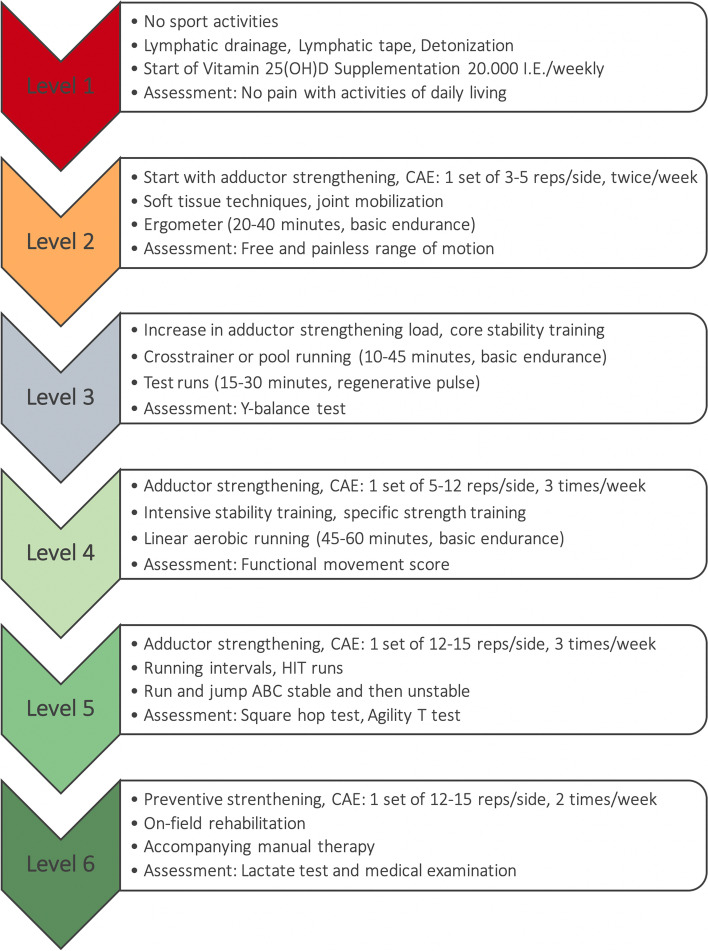
Rehabilitation Program

All injured youth soccer players with clinically and radiologically diagnosed long-standing pubic-related groin pain participated in an intensive 6-level rehabilitation program (Fig. 1). It was developed according to the evidence available for adult players and adapted to the specific characteristics of youth players, resulting in a step-wise return-to-play process for each individual youth player [10, 11, 13, 25, 33–40]. Except for the first phase, there was no time recommendation for completing the rehabilitation levels. Rather, a player’s progress through the levels was based on meeting specific criteria [41]. Progression to the next level in the program was possible as soon as the assessment test at the end of the current phase was passed and pain scores during therapy/activity and adductor squeeze tests were < 3 on the VAS (Visual Analog Scale). Values equivalent to the preliminary values scored during the preseason diagnostics (Y-balance test, functional movement test (FMS), square hop test, agility T test, lactate test) had to be achieved on the metric tests to advance to the next level. The full rehabilitation program can be seen in Additional file 1.

The ultimate return-to-play decision was made at the end of level 6 by the player and the medical staff after the player passed the final assessments (lactate test and medical examination). In the final medical examination of the pubis, the *M. rectus* abdominis and the adductors needed to be painless on palpation and the maximal isometric adduction at 0, 45 and 90° and adductor stretch needed to be painless. It was important that the player did not report any pain while passing, kicking or shooting the ball.

### Clinical outcome criteria

The primary outcome parameter for assessing the benefit of the standardized intensive conservative treatment program in this study was the consecutive time to return to play (RTP) accounting from the beginning of rehabilitation. Return to play was defined as the return to full participation in team training and availability for match selection [42]. The secondary outcome parameters were the recurrence of symptoms in the follow-up period and the clinical result at final follow-up two years after the return to play. Clinical outcome measures, as deducted from the current literature at the 24-month follow-up were: Pubic-related groin pain, pain of the rectus abdominis and the adductor insertions at the pubic bone; pain during active adduction in the adductor squeeze tests; groin pain during or after playing soccer; and reduction in the Tegner activity level [42] due to groin problems. If all four measures were negative, the result was labeled excellent; if three measures were negative, the result was good; if two measures were negative, the result was fair; and if one measure was negative, the result was poor.

### Statistical analysis

Statistical analysis and presentation of data were completed using IBM SPSS Statistics for Macintosh version 27.0 software (IBM, New York, USA). Descriptive statistics were used to provide an overview of clinical measures and the time to return to play. Continuous variables are reported as the means with standard deviations. Missing data from athletes being lost to follow-up were excluded. Differences between groups, to determine the risk factors, were evaluated by using multivariate logistic regression analysis. A multiple linear regression analysis and was used to access possible influencing factors on the time to return to play. Both regression models included age, team, age group within team, position, FMS score and MRI-grade. The level of significance was set at *P* < .05.

## Results

During the study period, a total of 14 out of 189 (incidence 7.4%) soccer players showed clinical and radiological signs consistent with long-standing pubic-related groin pain and required specific treatment based on the rehabilitation scheme. All players were completing follow-up, no data was missing. The average age of the players at the time of the injury was 16.1 ± 1.9 years. Two injured players played in U14, one in U15, two in U16, four in U17, three in U19 and two in U23. Nine of 14 players (64,3%) sustained their injury in the first half of the season. A total of 35.7% of the players had MRI grade 1, 50.0% had MRI grade 2 and 14.3% had MRI grade 3 edema according to the Krueger classification system [29].

Risk factor analysis showed a significant influence of age group within the team (*p* = .007). Only players in the younger age group were affected. No significant effect was observed of age, team, position or preinjury FMS score (Table 1).

**Table 1:** Risk factors for long-standing pubic-related groin-pain in young academy soccer players

	Study Group	Control Group	Significance
**n**	14 (7.4%)	175 (92.6%)	
**Age (y, mean ± SD)**	16.1 ± 1.9	15.2 ± 2.9	.139
**Team**	U14 *n* = 2/U15 *n* = 1/U16 *n* = 2/U17 *n* = 4/U19 *n* = 3/U23 n = 2	U12 *n* = 16/U13 *n* = 20/U14 *n* = 20/U15 *n* = 25/U16 n = 22/U17 n = 22/U19 *n* = 22/U23 *n* = 28	.744
**Age group within team (young/old)**	9/0	36/36	.007*
**Position**	GK *n* = 0/DF n = 3/ MF *n* = 8/FW *n* = 3	GK *n* = 17/DF *n* = 47/ MF *n* = 70/FW *n* = 41	.564
**FMS**	14.7 ± 1.6	15.3 ± 0.8	.698

*GK* goalkeeper, *DF* defender, *MF* midfielder, *FW* forward, *FMS* Functional Movement Score, * level of significance *p* < .05

The average time to return to play of all players was 135.3 ± 83.9 days. The time to return to play was significantly influenced by the severity of bone-marrow edema on MRI (*p* = .009). Players with MRI grade I edema returned to play after an average of 71.6 ± 24.1 days, whereas players with MRI edema grade 2 returned after 147.9 ± 81.9 days, and players with MRI grade 3 edema returned after 250.5 ± 30.4 days. No significant impact on time to return to play was found for age or preinjury FMS score (Table 2).

**Table 2:** Multiple linear regression showing the effect of age, FMS and grade on RTP

	Age	FMS	MRI grade
**Regression coefficient (± standard error)**	−15.1 ± 11.8	−6.7 ± 12.3	94.4 ± 26.7
**Significance**	.259	.609	.009*

*FMS* Functional Movement Score, * level of significance *p* < .05

Only one player suffered from a recurrence of nonspecific symptoms (7.1%) during the FU-period without pressure pain over the symphysis or bone marrow edema in the MRI. This was also a younger player within team. Conservative therapy was prolonged, resulting in a pain-free return to play two weeks later. Thus, the clinical outcome following conservative treatment for long-standing pubic-related groin pain pubis according to the presented scheme with 24-month FU was excellent in all 14 cases. No player had to reduce activity due to pubic-related groin-pain symptoms at the time of follow-up (Table 3).

**Table 3:** Outcome parameters

	n	RTP rate	RTP time (mean ± SD)	Recurrence of symptoms^a^	Outcome at follow-up
**Value**	14	100%	135.3 ± 83.9d	1 (7.1%)	14 (100%) excellent

*RTP* Return-to-play; ^a^ Pubic-related groin pain, pain of the rectus abdominis and the adductor insertions at the pubic bone; pain during active adduction in the adductor squeeze tests; groin pain during or after playing soccer; and reduction in the Tegner activity level due to groin problems

## Discussion

The most important finding in the present study was that a sustainable return to play with a minimal risk of reinjury can be expected after completing a standardized, supervised, criteria-based conservative treatment program for long-standing pubic-related groin pain in professional youth soccer players. After a mean of four and a half months, players returned to full participation in team training and were available for match selection. In the follow-up period of 24 months, no player had to reduce activity due to pubic-related groin pain symptoms.

The incidence of long-standing pubic-related groin pain in the youth academy was 7.4% in the present study. To date, epidemiological data regarding the incidence of long-standing pubic-related groin pain in youth soccer players is lacking. Studies including adult athletes, however, reported incidence rates of 0.5 to 8%, with the highest incidences in distance runners and athletes in kicking sports, especially male soccer players [11, 43]. This variance is likely attributable to the current absence of standardized diagnostic criteria for long-standing pubic-related groin pain, as well as actual variability between the different sports [43].

### Risk factors

Analyses of risk factors for long-standing pubic-related groin pain in soccer or other sports are also lacking. However, there is an abundance of data on groin and hip injuries, including long-standing pubic-related groin pain as one of the potential differential diagnoses [7, 15, 23, 44–48].

Pfirrmann et al. suggested that both an increased player load and height gain augment the risk of sustaining an overuse injury in this region [44]. A recent systematic review of risk factors in both youth and adult athletes (age range 12–41 years) found level 1 and 2 evidence that in particular, a higher level of play is associated with an increased risk of groin injury in sports [23]. Consistent with these findings, we found a significant influence of the age group within the team in our risk factor analysis. Only players in the younger age group were affected. This risk may result from a higher intensity of training and game play and a greater number of training hours [23, 37]. The fact that the injuries mainly occurred in the first half of the season, additionally supports this assumption. Previous research confirms that age itself is not a significant risk factor for groin injuries in soccer players [45–47, 49].

Regarding the influence of player position on the development of specific pubic-related groin pain symptoms, no significant effects were identified in the present study, which is consistent with the existing studies in the literature [37, 45, 50, 51]. Only one study observed elevated risks of groin or adductor injuries in goalkeepers [46]. This may be related to hip loading during lateral movements performed by goalkeepers and to repetitive traumas to the lateral hip region due to lateral diving [52]. Moreover, preinjury FMS score was not a significant risk factor for the development of long-standing pubic-related groin pain in the present study. This is consistent with the findings of the studies of Linek et al. and Bakken et al., in which the FMS score could also not be used to differentiate between healthy athletes and those complaining of hip/groin symptoms [53, 54]. In addition to our analyzed factors, Whittaker et al. found that previous groin injury, reduced hip abductor and adductor strength and lower levels of sport-specific training were relevant risk factors for groin injury in sports in their systematic review [23].

Furthermore, in this study, we analyzed the potential prognostic factors on MRI associated with delayed return to play. Pubic bone marrow oedema is quite frequent in both symptomatic and asymptomatic soccer players due the biomechanical stress in this region, nevertheless higher grades of pubic bone marrow oedema are significantly more present in symptomatic players than asymptomatic players [55]. In the present study, a significant association was noted between MRI grade and return to play. The duration of symptoms was comparable to the results reported by Krueger [29]. According to Krueger and the findings in this study, the symptoms of long-standing pubic-related groin-pain in players with grade 1 edema will last up to three months, while six and twelve months of absence from sports can be expected for players with grades 2 and 3, respectively. These perceptions might help clinicians counsel soccer athletes with diagnosed long-standing pubic-related groin pain about the expected time loss due to the injury. In a diagnostic study by Gaudino et al., the extent of bone marrow edema appeared not to be important as a prognostic factor, but a significant association was observed between a more elevated signal intensity within the bone marrow measured on STIR images, which is considered to be indicative of bone marrow edema, and partial recovery [12]. Since all athletes in the present study achieved complete recovery during follow-up, the aforementioned findings are not supported by this study.

### Rehabilitation

Long-standing pubic-related groin pain is typically described as a self-limiting condition that responds well to conservative therapy [11, 13, 26, 56]. The primary therapeutic goal in youth soccer is a rapid but sustainable return to play without recurrent problems and no further negative influence of the injury on the athlete’s career. Undergoing extended periods of rehabilitation is challenging, especially for professional youth athletes and those surrounding them, including trainers, therapists, player agents and family members. For this reason, an early and exact diagnosis and a time-effective therapeutic approach are needed. To the best of our knowledge, this is the first study examining a conservative rehabilitation protocol for long-standing pubic-related groin pain in professional youth players. The existing evidence is therefore limited to adult athletes. Different conservative therapeutic approaches have been described, although it has not been clarified which approach has the most beneficial effect on the healing process [11]. Few studies with level one evidence have been published recently, and most of the scientific articles are retrospective case series or case reports, making it difficult to draw conclusions. Nevertheless, Schöberl et al. showed the superiority of intensive conservative therapy over the absolute abstention from sports in their level 1 study [10]. Recent studies have emphasized the importance of an individualized, progressive, multimodal rehabilitation program [10, 11, 25, 36]. In the program presented in this study, patients move through the levels after they are able to perform exercises without pain and achieve adequate levels of motion and core stability grading [11]. Accurate assessment allow alterations of the athlete’s schedule, which may be necessary to allow the pubic bone and tendinoperiosteal structures suitable time for adaptation [25].

Because strengthening hip adductors plays an important role in reducing the prevalence and rate of groin injuries in soccer, a level-based adductor strengthening program including the Copenhagen adduction exercises (CAE) was integrated into the rehabilitation protocol [33]. Shock wave therapy can be used in addition to physical exercise, and the results are promising [10]. Nevertheless, shock wave therapy was not used with our athletes due to concerns regarding patient safety, given that growth plate closure can continue up to the age of 16 years. Few articles have been published about steroid injection therapy, with insufficient evidence regarding the short- and long-term efficacy [11, 39, 57]. Our greatest concern, which kept us from using corticoids in youth soccer players, was the risk of tendon degeneration or even rupture, which has been described in high-level studies of other tendon attachments [58, 59]. A different autologous biological approach that would not cause further harm to the tendons might be ultrasound-guided platelet-rich plasma (PRP) injections. Currently, only one case report reporting promising results after PRP injections for distal rectus abdominis tendinopathy and long-standing pubic-related groin-pain is available [60].

As suggested by Hopp et al. surgery should be reserved for a limited subgroup of patients who fail to improve with conservative management involving at least 12 months of a well-conducted rehabilitative program [14, 27].

Many different surgical techniques have been described, but the majority of published studies have a low level of evidence, and no randomized controlled trials have been performed. Surgical neurolysis, open surgical resection, arthroscopic curettage and symphysis joint arthrodesis have been described as partially successful treatment options [11, 14, 27, 43].

### Return to play

Return to play times in cases of the conservative management of long-standing pubic-related groin pain vary due to differences in the types of sports, sports levels, clinical severities and treatment methods analyzed in the studies. Moreover, available data is limited to adult patients. Gaudino et al. retrospectively analyzed 24 professional premier league soccer players with long-standing pubic-related groin pain. All patients returned to high-level sports activity, but the time interval varied, with less than 3 months in 18 patients, 3–6 months in 5 patients and 6–12 months in one patient. Fifteen patients achieved a complete recovery, as measured by the absence of symptoms 18 months after the initial MRI, while 9 patients achieved a partial recovery, with persistent groin pain during particular movements [12]. Verall et al. described that only 63% of professional soccer players diagnosed with long-standing pubic-related groin pain returned within 5 months, and only 41% of these were asymptomatic [61]. The research group of Pajaanen et al. reported that 50% of nonoperative treated patients had disabling symptoms after 1-year follow-up and all of them stopped elite sport within 3 years [62]. The conservative rehabilitation program detailed within this study, may account for similar or even improved return to sport times and asymptomatic follow-up after 2 years and could be a template for other studies concerning conservative management of long-standing pubic-related groin-pain in professional youth soccer.

### Limitations

The limitations of this study are the lack of an adequate control group and the relatively small patient cohort. Moreover, other possible relevant risk factors like height-gain, BMI and amount of exposure to matches and training were not collected in our study. The presence of pubic apophysistis as described by Sailly et al. could not be accurately described in our patient cohort, as we used 1.5 T MRT to avoid radiation on the gonadal region of the adolescent players [16].

However, all patients were professional youth soccer players from a first division soccer club; thus, after applying strict diagnostic criteria, they received a standardized treatment schedule. Further prospective studies including larger cohorts and longer follow-up periods are needed. To our knowledge, no study has investigated the results of a conservative rehabilitation program for long-standing pubic-related groin-pain in youth soccer players. In this study, we were able to describe a return-to-play time that was comparable to that in adult players, and most importantly, we showed a low rate of recurrence and excellent outcomes at the 24-month follow-up, which is crucial, especially in youth athletes ready to advance to adult professional soccer.

## Conclusion

Long-standing pubic-related groin-pain is an overuse entity in youth soccer players that results in a relevant loss of training and play time. Being part of the younger age group within the team is a significant risk factor. To avoid overuse, training load should be precisely monitored in these players. Almost all injured players returned to play without recurrent symptoms after participating in the criteria-based conservative rehabilitation program.

## Supplementary Information

**Additional file 1.**

## Data Availability

The data which analyzed during the study are stored in our hospital and are available from the corresponding author on reasonable request.

## Abbreviations

CAE: Copenhagen adduction exercise
FMS: Functional movement screen
HIT: High intensity training
MRI: Magnetic resonance imaging
PRP: Platelet-rich plasma
RTP: Return to play
RTS: Return to sports
STIR: Short tau inversion recovery
