# Supplementary material for: Long-standing pubic-related groin pain in professional academy soccer players: a prospective cohort study on possible risk factors, rehabilitation and return to play
Source: BMC Musculoskelet Disord. 2021 Nov 17;22:958. doi: 10.1186/s12891-021-04837-x (PMC8600924; doi:10.1186/s12891-021-04837-x)
Supplement: Supplementary file 1 — Additional file 1. [file 12891_2021_4837_MOESM1_ESM.docx]

**Appendix**

**Rehabilitation Program**

*Level 1:*

For the first 7-14 days, corresponding to level I of the standardized schedule, no sports activities were permitted. Players received lymphatic drainage, lymphatic tapes and trigger point therapy of the adductors and the rectus abdominis with an appropriate distance to the lesion for 60 min at least three times a week to achieve detonization of the muscle groups inserting at the os pubis and to reduce the players’ symptoms. According to the available data, the concentration of serum vitamin 25(OH)D should exceed 40 ng/mL in youth players. At this concentration, the vitamin exerts a beneficial effect on muscle tissue and reduces the frequency of stress damage to bone tissue.^9,54^ Therefore, 40 ng/mL was selected as the minimum target level during therapy, and vitamin D was supplemented at 20.000 I.E. weekly for athletes with serum vitamin 25(OH)D deficiency.

*Level 2:*

The adductor strengthening program, including the Copenhagen adduction exercises (CAE), was an integral part of our rehabilitation protocol and started in level 2, with only 3-5 repetitions 3 times per week, as described by Harøy et al.^20^ Progression in these concentric, eccentric and isometric exercises was only undertaken when the VAS pain scores were < 3. Myofascial release and mobilizations of the knee, hip joint, SI joint and lumbar spine (if necessary) were begun, and cycling on an ergometer was introduced as cardiovascular training. Moreover, gentle, prolonged stretching, except for the adductors and ischiopubic muscles, was performed.

*Level 3:*

In addition to the adductor strengthening program, abdominal core isometrics targeting the transversus abdominis, abdominal crunches, gluteal bridges with and without resistance bands, Swiss ball exercises for the abdominal core, leg swings, standing hip adduction, standing hip circles, hip flexion, trunk rotation, tension arc performed with elastic bands and one-leg coordination exercises were started in level 3 to improve lumbo-pelvic stability. Workouts on the crosstrainer and pool running were incorporated as cardiovascular training. Rapid stop-and-go movements were avoided by performing only linear test runs of 15-30 minutes with VAS pain scores < 3.

*Level 4:*

In the fourth level, adductor strengthening was further intensified with regard to eccentric load and number of repetitions. In addition, stability training and gym-based strength training were increased. Longer aerobic runs of 45-60 minutes were performed. Running was gradually increased, and changes in pace and direction were introduced. If VAS pain scores were > 3 during or after the activity, active training was paused for a day. The following two days, the players trained with reduced intensity and returned to the former intensity on the third day.

*Level 5:*

In addition to a further increase in the intensity of adductor strengthening in Level 5, multidirectional movement activities were started. Initially, this involved running ABCs, including curve and slalom run variations. The curve and slalom angles were varied to include sharper curvature runs, thereby increasing the amount of loading on the adductors. Once a good baseline of movement mechanics had been achieved, full cutting and change of direction at increased intensities were undertaken, and unstable undergrounds were used for proprioceptive exercises. Unpredictable movements, opponent drills, and contact drills were incorporated into the end stage of rehabilitation, after which players progressed to on-field rehabilitation.

*Level 6:*

In Level 6, soccer-specific training was introduced on the field with ball work and more dynamic ball striking, from half volleys to full volleys and to passing over various distances. Additionally, players partially participated in selected team training sessions at this level. Intensive manual therapy and preventive adductor strengthening training were continued beyond the time of return to play.
